# Area-weighted unipolar voltage to predict heart failure outcomes in patients with ischaemic cardiomyopathy and ventricular tachycardia

**DOI:** 10.1093/europace/euad346

**Published:** 2024-02-03

**Authors:** Robert Rademaker, Yoshi Kimura, Marta de Riva Silva, Hans C Beukers, Sebastiaan R D Piers, Adrianus P Wijnmaalen, Olaf M Dekkers, Katja Zeppenfeld

**Affiliations:** Department of Cardiology (C-05-P), Leiden University Medical Center, P.O. Box 9600, Albinusdreef 2, 2333 ZA, Leiden, The Netherlands; Willem Einthoven Center of Arrhythmia Research and Management, Leiden, The Netherlands; Department of Cardiology (C-05-P), Leiden University Medical Center, P.O. Box 9600, Albinusdreef 2, 2333 ZA, Leiden, The Netherlands; Willem Einthoven Center of Arrhythmia Research and Management, Leiden, The Netherlands; Department of Cardiology (C-05-P), Leiden University Medical Center, P.O. Box 9600, Albinusdreef 2, 2333 ZA, Leiden, The Netherlands; Willem Einthoven Center of Arrhythmia Research and Management, Leiden, The Netherlands; Department of Cardiology (C-05-P), Leiden University Medical Center, P.O. Box 9600, Albinusdreef 2, 2333 ZA, Leiden, The Netherlands; Department of Cardiology (C-05-P), Leiden University Medical Center, P.O. Box 9600, Albinusdreef 2, 2333 ZA, Leiden, The Netherlands; Willem Einthoven Center of Arrhythmia Research and Management, Leiden, The Netherlands; Department of Cardiology (C-05-P), Leiden University Medical Center, P.O. Box 9600, Albinusdreef 2, 2333 ZA, Leiden, The Netherlands; Willem Einthoven Center of Arrhythmia Research and Management, Leiden, The Netherlands; Department of Clinical Epidemiology, Leiden University Medical Center, Leiden, The Netherlands; Department of Cardiology (C-05-P), Leiden University Medical Center, P.O. Box 9600, Albinusdreef 2, 2333 ZA, Leiden, The Netherlands; Willem Einthoven Center of Arrhythmia Research and Management, Leiden, The Netherlands

**Keywords:** Ischaemic cardiomyopathy, Electroanatomical mapping, Heart failure, Unipolar voltage, Ventricular tachycardia

## Abstract

**Aims:**

Patients with ischaemic cardiomyopathy (ICM) referred for catheter ablation of ventricular tachycardia (VT) are at risk for end-stage heart failure (HF) due to adverse remodelling. Local unipolar voltages (UV) decrease with loss of viable myocardium. A UV parameter reflecting global viable myocardium may predict prognosis. We evaluate if a newly proposed parameter, area-weighted unipolar voltage (awUV), can predict HF-related outcomes [HFO; HF death/left ventricular (LV) assist device/heart transplant] in ICM.

**Methods and results:**

From endocardial voltage maps of consecutive patients with ICM referred for VT ablation, awUV was calculated by weighted interpolation of local UV. Associations between clinical and mapping parameters and HFO were evaluated and validated in a second cohort. The derivation cohort consisted of 90 patients [age 68 ±8 years; LV ejection fraction (LVEF) 35% interquartile range (IQR) (24–40)] and validation cohort of 60 patients [age 67 ± 9, LVEF 39% IQR (29–45)]. In the derivation cohort, during a median follow-up of 45 months [IQR (34–83)], 36 (43%) patients died and 23 (26%) had HFO. Patients with HFO had lower awUV [4.51 IQR (3.69–5.31) vs. 7.03 IQR (6.08–9.2), *P* < 0.001]. A reduction in awUV [optimal awUV (5.58) cut-off determined by receiver operating characteristics analysis] was a strong predictor of HFO (3-year HFO survival 97% vs. 57%). The cut-off value was confirmed in the validation cohort (2-year HFO-free survival 96% vs. 60%).

**Conclusion:**

The newly proposed parameter awUV, easily available from routine voltage mapping, may be useful at identifying ICM patients at high risk for HFO.

## Graphical Abstract

**Graphical Abstract:**
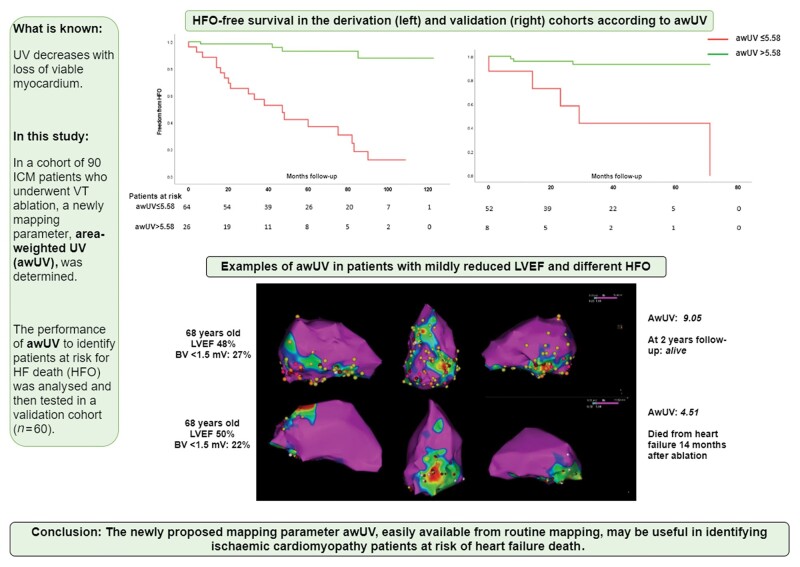

**What’s new?**

- Area-weighted unipolar voltage (awUV) corrects for (over)sampling bias of the scar area in unipolar voltage mapping.
- Area-weighted unipolar voltage is easily available from routine mapping during standard procedures.
- A critical reduction of awUV, as surrogate of global viable myocardium in the left ventricle, provides important prognostic information on heart failure–related deaths in ischaemic cardiomyopathy patients followed after catheter ablation of ventricular tachycardia.

## Introduction

Patients with ischaemic cardiomyopathy (ICM) and ventricular tachycardia (VT) are at risk of progressive heart failure (HF) and HF death.^1,2^ Ablation of VT is an important treatment option in patients with ICM and VT.^3,4^ Prediction of rapid development of HF death in patients undergoing VT ablation is important for patient management after ablation.^5^ Multiparameter risk scores have been developed to predict outcome in mixed cohorts of patients with HF and have been applied in patients with non-ischaemic cardiomyopathy (NICM) undergoing VT ablation.^6,7^ Data on HF outcome prediction in patients with ICM undergoing VT ablation are scarce.

Left ventricular ejection fraction (LVEF) and changes in LVEF over time are important predictors of all-cause mortality in ICM and NICM.^8–11^ Independently from LVEF, myocardial fibrosis, both compact and diffuse and its quantification, is increasingly recognized as prognostic marker in ICM and NICM^12–14^ Electroanatomical voltage mapping is considered the invasive gold standard to identify loss of viable myocardium replaced by fibrosis.

The size of low unipolar voltage areas (LVA) could identify NICM patients with irreversible LVEF impairment.^15^ Different unipolar voltage (UV) cut-off values have been proposed to define LVA.^16–18^ However, studies in NICM patients have shown a strong inverse linear relationship between UV and the amount of diffuse fibrosis.^19^ As a consequence, a single cut-off value cannot quantify variable degrees of diffuse fibrosis. In addition, the size of LVA, measured by tools provided by 3D mapping systems, is influenced by homogeneity and density of mapping, which is prone to sampling bias towards the dense infarct areas.

Here, we propose a novel method, area-weighted unipolar voltage (awUV), as surrogate for the total amount of remaining viable myocardium in patients with ICM and VT. We hypothesized that a critical reduction of awUV identifies patients at risk for worse HF-related outcome (HFO) after VT ablation, including death/LV assist device implantation (LVAD)/heart transplant (HT), and improves currently available clinical risk stratification.

## Methods

### Patient population and study design

The population consisted of consecutive patients with ICM who underwent radiofrequency catheter ablation (RFCA) for sustained VT at the Leiden University Medical Center from January 2012 to September 2018. Patients were subdivided into derivation and validation cohorts according to the procedure date (derivation cohort, January 2012–January 2016; validation cohort, February 2016–September 2018). The endpoint of inclusion in the validation cohort was chosen to allow for appropriate follow-up time and the assumption that a sufficient number of patients will have undergone ablation during the shorter period considering the increasing number of catheter ablation (CA) performed in our centre since 2016. Diagnosis of ICM was based on the presence of wall motion abnormalities on echocardiography, non-reversible perfusion defects, or the presence of transmural/subendocardial late gadolinium enhancement (LGE) in the perfusion area of a coronary artery with significant stenosis. All patients were treated according to a routine clinical protocol and provided informed consent for the procedure. No changes in the clinical or RFCA protocols were made between January 2012 and September 2018. The study protocol was approved by the local ethics board (METc).

### Pre-procedural analysis

All patients underwent clinical evaluation prior to the procedure, consisting of a medical history, physical examination, routine lab testing, and echocardiography. Kidney disease was defined as estimated glomerular filtration rate of <45 mL/min/1.73 m^2^. Left ventricular ejection fraction was determined using the Simpson bi-plane method. Patient records were reviewed for documentation of (clinical) VTs. All anti-arrhythmic drugs, with the exception of amiodarone, were discontinued for five half-time lives in stable patients before the ablation procedure. All post-infarct patients who are undergoing VT ablation in our centre are evaluated for residual ischaemia and undergo revascularization during admission in case of significant residual or new stenosis of the coronary arteries before mapping and ablation.

### Electroanatomical voltage mapping and ablation

Procedures were performed under conscious sedation or general anaesthesia when deemed necessary. Mapping of the aortic root/cusps and endocardial electroanatomical mapping (EAM) of the LV was performed using a retrograde aortic and if needed a transseptal approach during sinus- or right ventricular (RV)–paced rhythm, with a 3.5 mm irrigated-tip catheter (Navistar Thermocool or Thermocool Smarttouch SF, Biosense Webster Inc. Diamond Bar, CA) and the CARTO3 system. The fill threshold was set at ≤10 mm. Electrograms were filtered at 30–400 Hz for bipolar and 1–240 Hz for unipolar recordings and displayed at 200 mm/s sweep speed. For the purpose of the analysis, LVA were defined as confluent areas with bipolar voltage (BV) ≤ 1.5 mV or UV ≤ 8.27 mV, respectively.^17,18^ Confluent areas with BV < 0.5 mV were considered dense scar. Ablation target sites were identified based on functional substrate mapping and, in addition, based on activation/entrainment mapping if VTs were tolerated, as previously described.^20^ Radiofrequency energy was delivered between 35 and 50 W with a limited temperature of 43°C and flow rate of 20–30 mL/min until pacing with high output did not capture (10 mA/2 ms). The induction protocol at the end of the procedure consisted of burst pacing and stimulation with four drive cycle lengths (600, 500, 400, and 350 ms) up to four extrastimuli from two RV sites and at least one LV site.

### Follow-up

Patients were followed at 3 and 6 months after the procedure and every 6 months thereafter. Follow-up included a medical history and implantable cardiac defibrillator (ICD) interrogation. The endpoint was the occurrence of adverse HFO, including death due to HF, LVAD implantation, or HT. For patients not followed at our institution, the referring physician was contacted for death, cause of death, LVAD implantation, or HT.

### Electroanatomical mapping analysis

All mapping data were reviewed off-line. For each point, the mapping window-of-interest was manually adapted to exclude far-field electrograms, artefacts, and injury current from the peak-to-peak measurement of BV and UV amplitudes. The aortic and mitral valve areas were identified and removed from the map to determine the true endocardial surface. Low-voltage areas (UV < 8.27 mV and BV < 1.5 mV) were manually measured and expressed as percentage of the total endocardial LV surface. Left ventricular volume was estimated by the CARTO software.

### Area-weighted unipolar and bipolar voltages

After manual correction and removing of the aortic and mitral valve areas, the endocardial EAM data and 3D meshes were transferred from CARTO to ParaView 3D visualization software version 5.7 (Kitware Inc., Clifton Park, NY) using custom made Python plugins. Area and area-weighted bipolar and unipolar voltages (awBV, awUV) were calculated as previously described.^21^ Briefly, (i) BV and UV values at each location on the LV endocardial surface mesh were determined by interpolating the voltages of the surrounding measured EAM contact points. The interpolation uses the weighted average of the EAM points within a specified radius and a Gaussian distribution to assign the weights. This interpolation ensures that adjacent points contribute more to the interpolated voltages than distant points. (ii) The total weighted BV and UV were calculated by mathematically integrating the BV and UV over the LV surface. For a detailed description on the calculations of area-weighted voltages, see Supplementary material online, *Figure S2*.

### Statistical analysis

Dichotomous and categorical variables are shown as number (%). Continuous variables are shown as mean ± standard deviation (SD) or median and interquartile range (IQR) depending on distribution. Variables were compared using an unpaired *t*-test or the Mann–Whitney *U* test when appropriate. Categorical variables were compared using a χ^2^ or the Fisher’s exact test. The difference was deemed statistically significant if the *P*-value was <0.05. Univariable Cox proportional hazard analysis was used to test the association between HFO (freedom from HFO) and baseline covariables. Propensity scores were calculated using univariable Cox regression and used for the calculations of likelihood ratios (LR). Propensity scores reflect a conditional probability of an assignment to a particular outcome given a vector of covariates. The propensity score is calculated for each patient on a 0–1 scale and is used, among other uses, as a representation of the conditional probability of an outcome given the observed covariates.^22^ Age, gender, and all clinical variables associated with HFO in univariable analysis (kidney disease, QRS duration, LVEF, and amiodarone use pre-study) were used to calculate the propensity score. Using these propensity scores, LR calculations were performed using the following steps: first, the base propensity score for the outcome was calculated. Then, each of the following variables was added in a separate model: UV LVA, awBV, and awUV. Each of these models contained two variables (propensity base score and the to be tested variable). The LR in comparison to the base model was calculated. The increased LR was significant if the LR χ^2^ test showed a *P*-value below 0.05. If that was the case, the added variable was deemed an improvement over the base model.

Using a receiver operating characteristics (ROC) curve, the optimal cut-off value associated with HFO was calculated for LVEF and the mapping-based parameters (UV LVA, BV LVA, awBV, awUV). The optimal cut-off selection was based on determining the maximal sum of sensitivity and specificity associated with the HFO.

The performance of the cut-offs derived from the derivation cohort was tested in the validation cohort using the Kaplan–Meier method and log-rank test.

Statistical analyses were performed using either SPSS version 27 (IBM, New Orchard Road, NY) or Stata version 16 (StataCorp LLC, Lakeway Drive, TX).

## Results

### Derivation cohort

The derivation cohort consisted of 90 consecutive patients [age 68 ± 8 years; 77 (86%) males; kidney disease: (32%); QRS duration 117 ms (95–146); LVEF 35% (IQR 24–40)], ablated in the predefined period. Median clinical VT cycle length (VTCL) was 352 ms (IQR 305–431), and 36 (40%) patients used amiodarone during mapping. The median LV volume was 213 cm^3^ (IQR 163–277). The mean LV surface and the median UV LVA were 194 ± 50 cm^2^ and 64% (IQR 47–85), respectively. Median awUV was 6.6 (IQR 5.2–8.6) and median awBV was 2.7 (IQR 2.1–3.5).

In the derivation cohort, 38 patients (42%) were rendered non-inducible for any VT, 48 patients (53%) had non-clinical VTs still inducible, and 4 (5%) patients remained inducible for the clinical VT, defined as procedural failure. Baseline characteristics, procedural outcomes, and mapping-derived data are provided in *Tables 1* and *2*.

**Table 1:** Baseline characteristics in derivation and validation cohorts

	Derivation cohort *N* = 90	Validation cohort *N* = 60	*P*-value
Age	68 ± 8	67 ± 9	0.32
Male	77 (86)	53 (88)	0.62
Hypertension	31 (34)	22 (37)	0.78
Diabetes mellitus	13 (14)	9 (15)	0.93
History of AF	27 (30)	13 (22)	0.26
Anterior MI	34 (38)	12 (20)	0.02
QRS duration, ms	117 (95–146)	109 (95–143)	0.70
LVEF, %	35 (24–40)	39 (29–45)	0.03
ICD before ablation	69 (77)	44 (73)	0.64
Prior PCI	35 (40)	30 (50)	0.47
Prior CABG	32 (37)	15 (25)	0.36
Medications at admission			
ACE inhibitor/ARB	74 (82)	47 (79)	0.96
Beta-blockers	64 (71)	52 (87)	0.03
Amiodarone	36 (40)	23 (38)	0.84
Medications at discharge			
ACE inhibitor/ARB	74 (82)	48 (80)	0.89
Beta-blockers	55 (63)	58 (97)	0.001
Amiodarone	39 (43)	20 (33)	0.22
VT clinical presentation			
Clinical VT cycle length, ms	352 (305–431)	375 (307–428)	0.50
Ablation outcomes			
Non-inducible after ablation	38 (42)	28 (45)	0.79
Non-clinical VTs inducible	48 (53)	30 (52)	0.96
Procedural failure	4 (5)	2 (3)	0.48

Results are shown as number (%), mean (SD), or median (IQR).

ACE, angiotensin converting enzyme; AF, atrial fibrillation; ARB, angiotensin receptor blocker; CABG, coronary artery bypass grafting; ICD, intracardiac defibrillator; LVEF, left ventricular ejection fraction; MI, myocardial infarction; PCI, percutaneous coronary intervention; VT, ventricular tachycardia.

**Table 2:** Mapping-derived data in derivation and validation cohorts

	Derivation cohort *n* = 90	Validation cohort *n* = 60	*P*-value
Number of points	245 ± 75	340 ± 281	0.08
Surface, cm^2^	194 ± 50	207 ± 52	0.90
LV volume, cm^3^	213 (163–277)	247 (188–326)	0.01
LVA UV < 8.27 mV, %	64 (47–85)	57 (43–73)	0.10
LVA BV < 1.5 mV, %	33 (20–41)	32 (20–40)	0.96
awBV	2.7 (2.1–3.5)	2.9 (2.5–3.3)	0.36
awUV	6.6 (5.2–8.6)	7.2 (6.3–8.8)	0.04

Results are shown as number (%), mean (SD), or median (IQR).

awBV, area-weighted bipolar voltage; awUV, area-weighted unipolar voltage; BV, bipolar voltage; LV, left ventricle; LVA, low-voltage area; mV, millivolt; UV, unipolar voltage.

### Follow-up

During a median follow-up of 45 months (IQR 34–83), a total of 36 (43%) patients died, 20 (22%) due to HF, 2 (2%) due to sudden cardiac death, and 14 (16%) due to a non-cardiac death. Twenty-three (26%) patients reached the HFO endpoint (20 deaths due to HF, 2 LVAD implantations, 1 HT) with a median time to HFO of 33 months (IQR 14−60). Twenty-nine (32%) patients had VT recurrence during follow-up. Causes of non-cardiac death are provided in Supplementary material online, *Table S1*.

### Clinical characteristics and prediction of outcome according to heart failure–related outcomes

In the derivation cohort, patients with HFO, compared with those without an event, were more likely to be female and had a lower LVEF, more often kidney disease, longer QRS duration, and more amiodarone use (see Supplementary material online, *Table S2*). Cox proportional hazards regression analysis showed that female gender, kidney disease, longer QRS duration, a lower LVEF, and amiodarone use pre-study were associated with HFO [LVEF, per 1% decrease: hazard ratio (HR) 1.10 (1.05–1.15); kidney disease: HR 4.82 (2.03–11.40); amiodarone use pre-study: HR 8.36 (3.07–22.73); male gender: HR 0.31 (0.13–0.77), QRS duration, per 1 ms increase: (1.01–1.04), all *P* < 0.01] (*Table 3*).

**Table 3:** Univariable analysis for HFO in derivation cohort

	HR	95% CI	*P*-value
Age (1 year increase)	1.03	0.98–1.08	0.24
Male	0.31	0.13–0.77	0.01
History of AF	1.48	0.64–3.43	0.36
Hypertension	0.99	0.41–2.43	0.99
Diabetes mellitus	0.92	0.27–3.12	0.90
Kidney disease	4.82	2.03–11.40	<0.001
Anterior MI	2.00	0.88–4.52	0.10
QRS duration (1 ms increase)	1.03	1.01–1.04	<0.001
LVEF (1% decrease)	1.10	1.05–1.15	<0.001
Amiodarone use pre-study	8.36	3.07–22.73	<0.001
LVA BV (1% increase)	1.05	1.02–1.07	<0.001
LVA UV (1% increase)	1.04	1.02–1.07	0.002
awBV (1 point decrease)	3.06	1.77–5.30	<0.001
awUV (1 point decrease)	2.09	1.54–2.83	<0.001

See *Tables 1* and *2* for abbreviations.

### Mapping data according to heart failure–related outcomes and prediction of outcome

Concerning the mapping-derived data, patients with HFO, when compared with patients without HFO, had a larger median unipolar and bipolar LVA and lower median awBV and awUV (see Supplementary material online, *Table S3*). In univariate Cox proportional hazard regression analysis, BV LVA, UV LVA, awBV, and awUV were all associated with HFO [BV LVA per 1% increase: HR 1.05 (1.02–1.07); UV LVA per 1% increase: HR 1.04 (1.02–1.07); awBV per 1 point decrease: HR 3.06 (1.77 −5.30); awUV per 1 point decrease: HR 2.09 (1.54–2.83), all *P* < 0.001, see *Table 3*].

Receiver operating characteristics analyses were performed to determine the optimal cut-offs for the HFO outcome. The awUV cut-off had a higher area under the curve (AUC) (cut-off 5.58, AUC 0.88) compared with the other parameters (cut-off and AUC: awBV, 2.85, 0.77; UV LVA, 71.6%, 0.80; BV LVA, 24.2%, 0.70; see Supplementary material online, *Figure S1*). The Kaplan–Meier survival estimates curve showed a significant difference in survival time when using the optimal awUV cut-off value (log-rank *P* < 0.001). Area-weighted unipolar voltage > 5.58 showed excellent discrimination between patients with HFO and patients without HFO [3-year HFO-free survival in awUV > 5.58: 40/41 (98%) vs. awUV < 5.58: 13/23 (57%)]. Kaplan–Meier survival curve is shown in the Graphical abstract.

### Goodness-of-fit tests

After adjustment for variables that influence the risk for HFO in a propensity score, awUV was the only mapping-derived parameter that significantly increased the likelihood ratio for HFO (LR χ^2^ 9.72, *P* = 0.002). Goodness-of-fit test results are displayed in *Table 4*.

**Table 4:** Goodness-of-fit tests: HFO

Derivation cohort	Likelihood ratio	*P*-value
Base propensity score	N.A.	N.A.
+UV LVA < 8.27 mV	2.06	0.15
+awBV	2.61	0.11
+awUV	9.72	0.002

See *Tables 1*, *2*, and *3* for abbreviations; propensity score parameters: age, male gender, kidney disease, LVEF, and QRS duration.

### Validation cohort

The validation cohort consisted of 60 patients [mean age 67 ± 9 years, median LVEF 39% (IQR 29–45)], and 23 (38%) of patients used amiodarone. The median clinical VTCL was 375 ms (IQR 307–428). Median LV volume was 247 cm^3^ (IQR 188–326), median LV surface 207 ± 52 ms, and median UV LVA 57% (IQR 43–73). Median awUV and awBV were 7.2 (IQR 6.3–8.8) and 2.9 (IQR 2.5–3.3).

In the validation cohort, 28 (45%) patients were rendered non-inducible after ablation, 30 (52%) of patients were inducible for a non-clinical VT, and 2 (3%) patients had procedural failure.

There were no significant differences in clinical characteristics between the validation and derivation cohorts, except a lower percentage of patients with anterior myocardial infarction (MI) (38% vs. 20%), more beta-blocker use pre-study (71% vs. 87%), and a slightly higher median LVEF [35% (IQR 24–40) vs. 39% (IQR 29–48)] in the derivation cohort (*Table 1*). The validation cohort had a larger median LV volume [213 (163–277) vs. 247 (188–326), *P* = 0.01], as determined by the 3D mapping system, which may be due to the slightly higher point density (245 ± 75 vs. 340 ± 281) and the related volume calculation algorithm of the 3D mapping system.

The awUV was higher [median 7.2 (IQR 6.3–8.8) vs. awUV 6.6 (IQR 5.2–8.6)]. The other mapping-derived parameters showed no significant difference (*Table 2*).

### Follow-up of validation cohort

During a median follow-up of 33 (IQR 17–50) months, eight patients (14%) reached the HFO endpoint, seven (12%) died due to HF, and one (2%) received an HT with a median time to HFO of 19 months (IQR 7–29). Five (8%) patients died due to non-cardiac causes, details are provided in Supplementary material online, *Table S1*.

### Validation of risk stratification

Applying the cut-off derived from the derivation cohort (awUV < 5.58) to the validation cohort, the Kaplan–Meier survival curve showed significant differences in HFO survival time. The cut-off provided excellent discrimination between patients at risk and patients who are not at risk for HFO. At 24 months follow-up, only 2/52 patients (4%) with awUV > 5.58 reached the HFO endpoint, compared with 3/8 patients (40%) with awUV < 5.58 (Graphical abstract).

## Discussion

This is the first study to evaluate the performance of a novel surrogate for the total amount of remaining viable myocardium, namely area-weighted unipolar voltage, to predict serious HF-related adverse events in patients with ischaemic heart disease undergoing CA for VT. Using this method, we could demonstrate that (i) reduced awUV was associated with HFO and was the only mapping-derived parameter with additional value in predicting HFO, after correction for known clinical predictors such as LVEF and kidney disease. (ii) Using the optimal cut-off of awUV > 5.58, patients at risk for HFO could accurately be identified in the derivation cohort and in a second independent validation cohort. The superiority in predicting HFO when compared with conventional LVA measurements suggests that awUV may be a better surrogate for the total amount of remaining viable myocardium, providing information of adverse remodelling in remote myocardium.

### Mortality in ischaemic cardiomyopathy with ventricular arrhythmia

In patients with ICM and VT referred for CA, mortality is high and the majority of patients dies due to deterioration of LVEF and subsequent HF.^23–25^ The high rates of HFO events in our derivation and validation cohorts (26% and 14%, respectively) are in line with this data.

Both the size of the compact post-infarct scar and the amount and extent of diffuse fibrosis as fundamental process of adverse remodelling of remote myocardium contribute to progressive HF and related adverse events in patients after MI.^26^

### Endocardial voltage mapping and outcome

Bipolar and unipolar voltages reflect the amount of excitable, viable myocardium in the vicinity of the recording electrode and may be helpful to delineate compact post-infarct scars and affected remote myocardium, contributing to risk stratification beyond LVEF.^19,27^ Prior studies have included the low bipolar voltage scar percentage together with LVEF, QRS duration, and VT storm in an electrophysiological risk score for death/HT for patients with NICM undergoing VT ablation.^28^ The ability of BV <1.5 mV to accurately delineate compact post-infarct transmural scars has been validated in animal infarct models.^29^ Unipolar voltage mapping is considered superior to detect diffuse fibrosis at any location, due to its wider field of view.^15,16,30^ With a cut-off of 32% of the total endocardial LV surface, UV LVA (<8.27 mV) could identify patients with irreversible NICM. In the same study, a UV LVA (<8.27 mV) of >145 cm^2^ was a strong predictor for cardiac mortality in a retrospective cohort of NICM patients.^31^ In a cohort of ICM patients, larger LVA (UV < 8.3 mV, > 45% of total LV endocardium) was associated with LV dysfunction.^32^ However, data on the prognostic value of the extent of the UV LVA for cardiac mortality and HFO in ICM patients are lacking.

### Superiority of area-weighted voltages compared with low-voltage area measurement

Of importance, conventional LVA measurement has several limitations. First, there is no general agreement on an optimal cut-off value to delineate areas of diseased myocardium within the LV in patients with ICM. The cut-off < 8.27 mV was derived from six young healthy subjects and may not be applicable for the remote myocardium in older post-MI patients and may overestimate the areas of LV affected by adverse remodelling.^17,18^ Applying this cut-off does not contribute to significant risk stratification for HFO in this study (LR calculations UV LVA <8.27 mV *P*-value = 0.15), and there was a significant size of low UV voltage (<8.27 mV) area in patients without HFO events (see Supplementary material online, *Table S3*).^16^

Both bipolar and unipolar voltages showed a linear relationship with the amount of viable myocardium in animal infarct models and humans.^33,34^ Any cut-off value cannot provide detailed information on the amount of viable myocardium within the area below or above the cut-off value.

Second, the LVA measurement performed with the tools provided by the 3D mapping system depends on the colour-coded interpolation of points influenced by the homogeneity and density of mapping points.

An accurate method to estimate the amount of viable myocardium by unipolar voltage mapping should include the absolute voltage amplitudes and should be able to correct for sampling bias towards the infarcted area and for the variable distance between points and their relative contribution to the total voltage.

To overcome these limitations, we have developed and validated the novel parameter, awUV. Weighted and interpolated voltages on the LV endocardial mesh are obtained using a Gaussian distribution, correcting for unevenly distributed mapping points (see Methods section and Supplementary material online for details). This study is the first to evaluate the prognostic value of this novel parameter LV endocardial awUV for HFO in patient with ICM referred for VT ablation.

### Area-weighted unipolar voltage as a measure of disease severity and prognosis

The newly proposed parameter may identify patients with a critical reduction of viable myocardium in the infarct area but, importantly, also in the remote myocardium. Transmural extent of the infarcted area has to reach 50% before dysfunction in contractability can be systematically identified.^35^ This could explain why a critical awUV decrease has additional prognostic value and may identify patients at risk despite an only mildly reduced LVEF at the time of mapping (Graphical abstract). Area-weighted unipolar voltage better predicted HFO than conventional LVA measurement. Adverse remodelling can progress over time, and a reduction of viable mass beyond a critical threshold, not obvious from the size of a LVA, may lead to more rapid progression of cardiac dysfunction.

Using the awUV cut-off (<5.58) derived from the derivation cohort, we could demonstrate a significant difference in HFO survival between critical awUV reduction patients and those above the optimal cut-off. The performance of the cut-off value could be validated in a second validation cohort. The results have important implications. Patients at high risk of rapid progressive HF may not only benefit from aggressive HF treatment but may be also considered for early screening for LVAD destination therapy or HT. Area-weighted unipolar voltage may further refine the risk stratification beyond LVEF in patients with ICM and VTs.^1,2^

Cardiac magnetic resonance imaging (MRI), using T1 mapping, can be considered as non-invasive gold standard for quantification of extracellular volume (ECV) as surrogate for remote adverse remodelling. A recent study has shown that in patients who underwent EAM for VT, there was a significant inverse relationship between LV endocardial voltage (both unipolar and bipolar) and ECV.^36,37^ However, the presence of ICDs still hampers whole heart MRI image acquisition due to the artefacts.^38^ In the cohort of this study, 75% of patients had an ICD implanted at the time of presentation. In addition, MRI acquisition is costly, not always available and an additional diagnostic modality. On the contrary, awUV is easily available in patients who undergo routine EAM for treatment of the presenting arrhythmia, with no additional cost or risks for the patient.

### Limitations

This study is a single-centre, observational, and retrospective study. Since the treatment with HF medication and anti-arrhythmic drugs was left to the treating cardiologist and electrophysiologist, it might have influenced HF progression in some patients.

A methodical limitation of this study may be the implementation of the only two-dimensional weighing of mapping points: the LV endocardial surface area. Studies have shown that wall thickness largely influences both unipolar and bipolar voltage.^19^ Incorporation of wall thickness would result in a three-dimensionally weighed voltage, which may increase accuracy even further for predicting adverse events and may result in a better prognostic tool. However, a previous study has found only a small range in observed wall thickness in post-MI VT ablation patients; accordingly, the effect of incorporating wall thickness might be limited.^16^ In addition, wall thinning may be part of the adverse remodelling and the field of view of the unipolar voltage is expected to exceed the wall thickness encountered in patients with ICM.^16,30^

The validation cohort is relatively small compared with the derivation cohort and the number of HFO lower than in the derivation cohort (23 vs. 8). However, we have decided to keep the predefined inclusion period of the validation cohort to allow for an appropriate follow-up period.

Previous data from our group could demonstrate the value of weighted UV to predict death from HF in NICM. However, post-infarct scars are fundamentally different from non-ischaemic fibrosis and post-infarct adverse remodelling is likely to differ from disease progression in NICM supporting separate analysis for different aetiologies.

As a whole, patients undergoing ablation for VT comprise a limited percentage of patients with ICM. However, we believe that our data are of value since there is limited data on prediction of HF outcomes in these patients.

## Conclusions

Area-weighted unipolar voltage is a newly proposed surrogate for LV viable myocardium, easily available through routine mapping and superior to LVA in predicting HFO. Because of its accuracy and incremental value to identify patients at risk for HF outcomes, it may refine current risk models and may be an important tool to identify patients who require early advanced HF management after RFCA of VT.

## Supplementary Material

euad346_Supplementary_Data

## Data Availability

The data underlying this article will be shared on reasonable request to the corresponding author.
