# Supplementary material for: Area-weighted unipolar voltage to predict heart failure outcomes in patients with ischaemic cardiomyopathy and ventricular tachycardia
Source: Europace. 2024 Feb 3;26(2):euad346. doi: 10.1093/europace/euad346 (PMC10838146; doi:10.1093/europace/euad346)
Supplement: euad346_Supplementary_Data [file euad346_supplementary_data.docx]

**Supplemental material:**

**Table S1. Causes of non-cardiac death**

|  | Derivation group | Validation group |
| --- | --- | --- |
| Surgical complications | 1 | 0 |
| Kidney disease | 1 | 0 |
| Malignancies | 3 | 1 |
| Pulmonary embolism | 1 | 0 |
| Pneumonia | 5 | 2 |
| Vasoplegic shock after ablation | 1 | 0 |
| Internal hemorraghing | 1 | 1 |
| Unknown | 1 | 1 |
|  |  |  |
|  |  |  |

**Table S2. Clinical characteristics according to HFO in derivation cohort**

See table 1,2 & 3 for abbreviations. Results are expressed as a number (%), mean ± SD, or median [IQR].

|  | Derivation cohort  N= 90 | HFO  N= 23 | No HFO  N=67 | P-value |
| --- | --- | --- | --- | --- |
| **Age** | 68±8 | 70±8 | 68±8 | 0.28 |
| **Male** | 77 (86) | 16 (70) | 61 (91) | 0.01 |
| **Hypertension** | 31 (34) | 16 (70) | 43 (64) | 0.64 |
| **Diabetes mellitus** | 13 (14) | 3 (13) | 10 (15) | 0.83 |
| **History of AF** | 27 (30) | 9 (39) | 18 (27) | 0.27 |
| **Kidney disease** | 29 (32) | 15 (65) | 14 (21) | <0.001 |
| **Anterior MI** | 34 (38) | 12 (52) | 22 (33) | 0.10 |
| **QRS-duration, ms** | 117 [95 – 146] | 158 [107 – 179] | 108 [93 -128] | 0.001 |
| **LVEF, %** | 35 [24 – 40] | 24 [16 – 35] | 38 [30 – 44] | 0.001 |
| **ICD before ablation** | 69 (77) | 21 (91) | 48 (72) | 0.05 |
| **Prior PCI** | 35 (40) | 8 (35) | 27 (42) | 0.54 |
| **Prior CABG** | 32 (37) | 5 (22) | 27 (42) | 0.08 |
| **Medications at admission** |  |  |  |  |
| ACE-inhibitor/ARB | 74 (82) | 16 (70) | 58 (87) | 0.07 |
| Beta-blockers | 64 (71) | 22 (96) | 42 (63) | 0.003 |
| Amiodarone | 36 (40) | 18 (78) | 18 (27) | <0.001 |
| **Medications at discharge** |  |  |  |  |
| ACE-inhibitor/ARB | 74 (82) | 17 (74) | 57 (86) | 0.17 |
| Beta-blockers | 55 (63) | 21 (91) | 34 (52) | 0.001 |
| Amiodarone | 39 (43) | 19 (83) | 20 (30) | <0.001 |
| **VT clinical presentation** |  |  |  |  |
| Clinical VT cycle length | 352 [305 – 431] | 375 [340 – 500] | 350 [300 – 415] | 0.001 |

**Table S3. Mapping derived data according to HFO**

|  | **Derivation cohort**  **N=90** | **HFO**  **N=23** | **No-HFO**  **N=67** | **P-value** |
| --- | --- | --- | --- | --- |
| **Mapping rhythm** |  |  |  |  |
| **Sinus** | 64 (71) | 13 (75) | 51 (76) | 0.08 |
| **AF** | 25 (28) | 9 (39) | 16 (24) | 0.08 |
| **RVP** | 1 (1) | 1 (4) | 0 (0) | 0.08 |
| **Number of points** | 245±75 | 221±49 | 246±73 | 0.74 |
| **Surface, cm^2^** | 194±50 | 252±82 | 186±48 | 0.003 |
| **LV-volume, cm^3^** | 213 [163 – 277] | 245 [222 – 372] | 188 [152 – 259] | 0.001 |
| **LVA UV <8.27mV, %** | 64 [47 – 85] | 91 [75 – 100] | 57 [40 – 76] | <0.001 |
| **LVA BV <1.5mV, %** | 33 [20 – 41] | 36 [30 – 45] | 27 [15 – 39] | 0.01 |
| **awBV** | 2.7 [2.1 – 3.5] | 1.95 [1.33 – 2.82] | 2.99 [2.36 – 3.73] | <0.001 |
| **awUV** | 6.6 [5.2 – 8.6] | 4.51 [3.69 – 5.31] | 7.03 [6.08 – 9.2] | <0.001 |

Results are expressed as a number (%), mean ± SD, or median [IQR].

See table 1, 2 & 3 for abbreviations. AF, atrial fibrillation; RVP, right ventricular pacing

**Figure S1: ROC curve different parameters to predict HFO in derivation cohort**
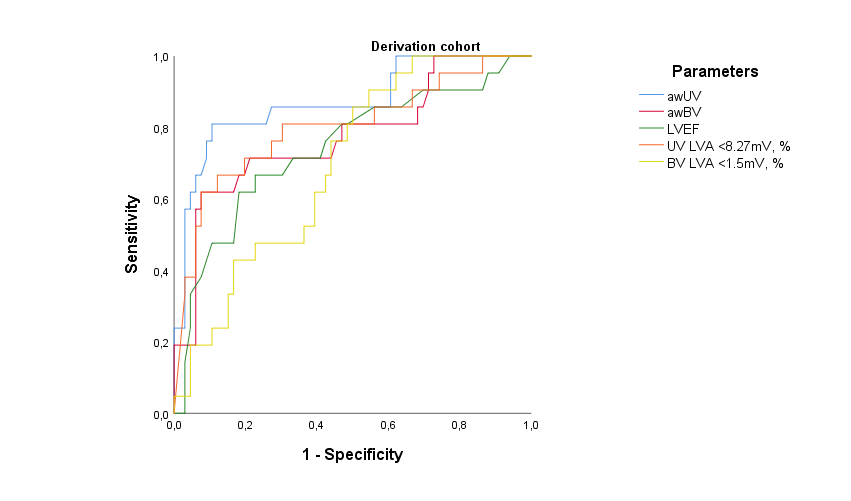


| **Parameter** | **Area under the curve** | **Optimal cut-off** |
| --- | --- | --- |
| ***awUV*** | 0.88 | 5.58 |
| ***awBV*** | 0.77 | 2.85 |
| ***LVEF*** | 0.74 | 32% |
| ***UV LVA <8.27mV*** | 0.80 | 71.6% |
| ***BV LVA <1.5mV*** | 0.70 | 24.2% |

See table 1 & 2 for abbreviations

***Area weighted bipolar and unipolar voltages***

After manual correction and removing of the aortic and mitral valve areas, the endocardial EAM data and 3D meshes were transferred from CARTO to ParaView 3D visualization software version 5.7 (Kitware Inc., Clifton Park, New York) using custom made Python plugins.(1) (Figure S2) To calculate area weighted bipolar and unipolar voltages (awBV, awUV) the following mathematical steps were performed:

(1) The BV and UV values at each location on the LV endocardial surface mesh were determined by interpolating the voltages of the surrounding measured EAM contact points. The interpolation corrects for differences of local mapping density and distances between points. The interpolation uses the weighted average of the EAM points within a specified radius and a Gaussian distribution to assign the weights. This interpolation ensures that adjacent points contribute more to the interpolated voltages than distant points. The weighting function for the Gaussian interpolation method is the Gaussian distribution: 𝑤(𝑑,𝜎) = 𝑎 exp (1/2 [𝑑/𝜎]2)

where 𝑑 is the distance from an EAM point to interpolated point, 𝜎 is the width of the distribution and 𝑎 is a normalization factor, ensuring that the sum of all weights is 1.

(2) The total weighted BV and UV were calculated by mathematically integrating the BV and UV over the LV surface. For this step, the interpolated value of each triangle of the mesh was multiplied by its surface area, and the result for all triangles were summed together. The area-weighted BV and UV (awBV and awUV) were determined by dividing the total weighted BV and UV by the total LV endocardial surface area.


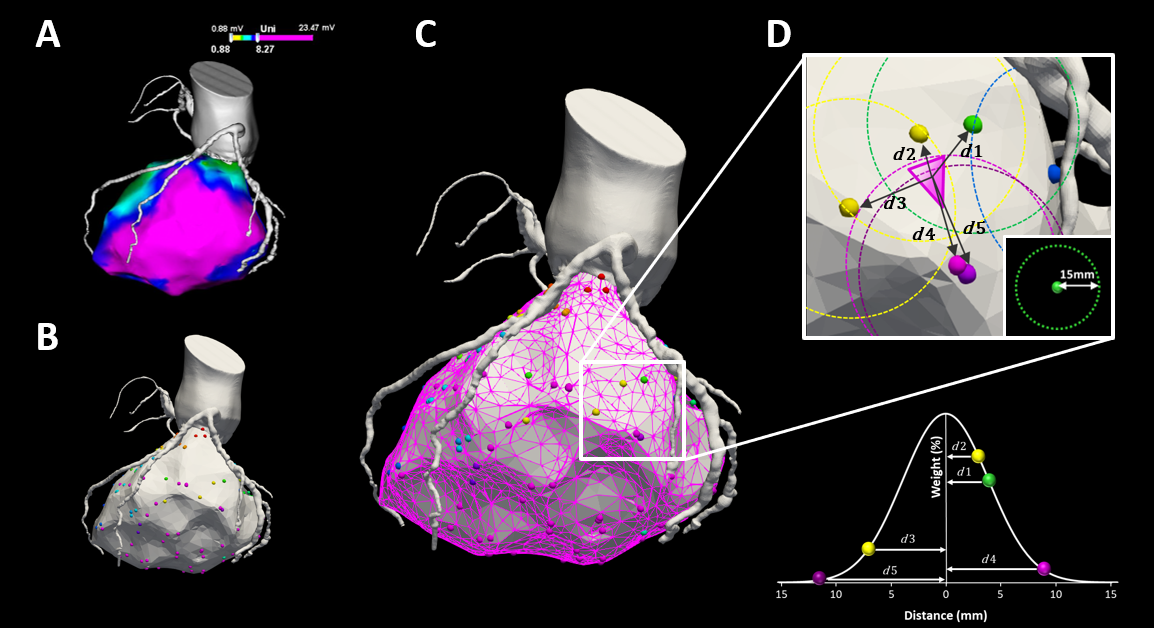
Figure S2

**Visualization of the calculation of area weighted voltages as described in Kimura et al (1).**

References

1. Kimura Y, Beukers HKC, Rademaker R, Chen HS, Ebert M, Jensen T, et al. Volume-Weighted Unipolar Voltage Predicts Heart Failure Mortality in Patients With Dilated Cardiomyopathy and Ventricular Arrhythmias. JACC Clin Electrophysiol. 2023;9(7 Pt 1):965-75.
